# Survival benefit of radiotherapy and nomogram for patients with primary tracheal malignant tumors: a propensity score-matched SEER database analysis

**DOI:** 10.1007/s00432-023-04896-8

**Published:** 2023-05-30

**Authors:** Zhen Zheng, Zhennan Du, Zhongjie Fang, Yunbin Shi, Xue Chen, Ming Jin, Kaitai Liu

**Affiliations:** 1 Department of Radiation Oncology and Chemotherapy, The Affiliated Lihuili Hospital of Ningbo University, Ningbo, 315000 China; 2 Department of Cardiothoracic Surgery, The Affiliated Lihuili Hospital of Ningbo University, Ningbo, 315000 China; 3 Department of Otolaryngology Head and Neck Surgery, The Affiliated Lihuili Hospital of Ningbo University, Ningbo, 315000 China; 4 Department of Respiratory Medicine, Ningbo Yinzhou No. 2 Hospital, Ningbo, 315000 China

**Keywords:** Tracheal tumors, Radiation therapy, Propensity score matching, Prognostic outcomes, Nomogram

## Abstract

**Purpose:**

The purpose of this study was to conduct a matched-pair analysis to assess the impact of radiotherapy (RT) on patients with malignant tracheal tumors using the surveillance, epidemiology, and end results database. Additionally, a predictive nomogram was developed for patients with malignant tracheal tumors.

**Methods:**

Propensity score matching (PSM) was used to minimize bias between the RT and no-RT groups. We utilized both univariate and multivariate Cox proportional hazards regression analyses to identify independent prognostic factors for patients and subgroups. We developed a novel nomogram and evaluated its results using the C-index.

**Results:**

A total of 648 patients between 1975 and 2019 were included, and 160 patients in RT were 1:1 propensity score-matched with no-RT. The independent prognostic factors for patients with tracheal malignant tumors were surgery, marital status, disease extension, pathology, and age. The independent risk factors for patients without surgery included RT and disease extension. The C-index confirmed that the nomogram accurately predicted the prognosis of patients with tracheal malignant tumors.

**Conclusions:**

Our findings suggest that RT may provide a survival benefit for tracheal cancer patients who did not undergo surgery. The nomogram can be a useful tool for predicting prognosis in patients with tracheal malignant tumors.

## Introduction

Primary tracheal tumors are an extremely rare type of cancer, accounting for less than 0.5% of all malignant tumors. According to population studies conducted in Finland, Denmark, the Netherlands, and England, it has been found that the annual incidence of tracheal cancer is around 0.1 per 100,000 individuals in the general population (Honings et al. 2007; Licht et al. 2001; Manninen et al. 1991; Nouraei et al. 2014). In adults, primary tracheal tumors are mainly malignant, with squamous cell carcinoma (SCC) accounting for approximately 50–66% of cases. Adenoid cystic carcinoma (ACC) is the second most common type, accounting for 10–15% of cases (Honings et al. 2007; Licht et al. 2001; Manninen et al. 1991; Urdaneta et al. 2011).

The primary treatment options for tracheal tumors include surgery and radiation therapy. Adjuvant postoperative radiotherapy (RT) is highly recommended for patients exhibiting high-risk characteristics, such as a positive margin. Small retrospective studies have actually shown improved outcomes in patients who received adjuvant RT (Graham 1997). Furthermore, 30–50% of patients have unresectable lesions at the time of diagnosis (Grillo and Mathisen 1990; Honings et al. 2009). There is limited data available to guide the treatment of unresectable tracheal tumors. However, some studies have shown that definitive RT has been used as a treatment option in patients who are not suitable candidates for resection. RT has also been used as local palliative therapy (Chow et al. 1993; Mornex et al. 1998). The existing literature on this subject is predominantly confined to small studies conducted at single institutions. To overcome this limitation, we conducted a matched-pair analysis using data derived from the Surveillance, Epidemiology and End Results (SEER) database. The objective of the study was to evaluate the effectiveness of radiotherapy as a management strategy for primary malignant tracheal tumors, whether they were resettable or unresectable.

## Materials and methods

### Patients

In this study, data were extracted from the SEER database spanning the period between 1975 and 2019, with the aim of examining patients diagnosed with malignant tracheal tumors. To be included in the study, patients had to meet the following criteria: (1) they were diagnosed with malignant tracheal tumors; (2) pertinent demographic variables, including age, sex, marital status, and the year of diagnosis, were available; and (3) clinical and pathological information, including pathology, disease stage, and treatment specifics, were available. The study ultimately included a cohort of 648 patients diagnosed with malignant tracheal tumors, of whom 443 were administered RT.

### Data collection

The study analyzed various variables to identify the risk factors associated with malignant tracheal tumors. These factors included age, marital status, sex, race, histology type, year of diagnosis, and disease extension. Survival analyses were also conducted to determine the prognostic factors for patients with primary malignant tracheal tumors. The study considered two treatment variables, namely, surgery and radiotherapy, based on the aforementioned factors. The primary outcome for this analysis was overall survival (OS), which was defined as the time interval between the day of diagnosis and the day of death for any reason.

### Propensity score matching (PSM)

In this study, propensity score matching (PSM) was utilized to address selection bias in patients who received RT. The propensity score in our study was computed as a probability score ranging from 0 to 1, which determined the likelihood of undergoing radiation therapy (RT) based on individual characteristics. The study used a logistic regression model to analyze and identify the independent correlations between all retrieved variables (*i*–*x*) and RT status (*xi*). Propensity score matching involved matching subjects in a 1:1 ratio without replacement with their nearest neighbor based on their propensity score by comparing survival outcomes between matched groups of RT and no-RT patients (Austin 2009, 2010). This approach helped to reduce potential biases in the patient selection process for those who received RT.

### Survival analysis

The present study utilized SPSS 26.0 and R software (version 4.2.3) for all statistical analyses, where a significance level of *P* < 0.05 (two-sided) was considered statistically significant. The study utilized the Kaplan–Meier method to estimate the overall survival rates for patients with varying lengths of follow-up. The log-rank test was applied to compare the overall survival curves of different groups. To identify potential risk factors in patients with primary malignant tracheal tumors, univariate logistic analysis was performed. Variables with a *P* value of less than 0.05 were further included in the multivariate logistic analysis using the “Forward LR” method in SPSS 26.0. Furthermore, a novel diagnostic nomogram was constructed using the “rms” package in R software (version 4.2.3) based on the independent risk factors.

## Results

### Selection of study cohort and propensity score matching

**Fig. 1:**
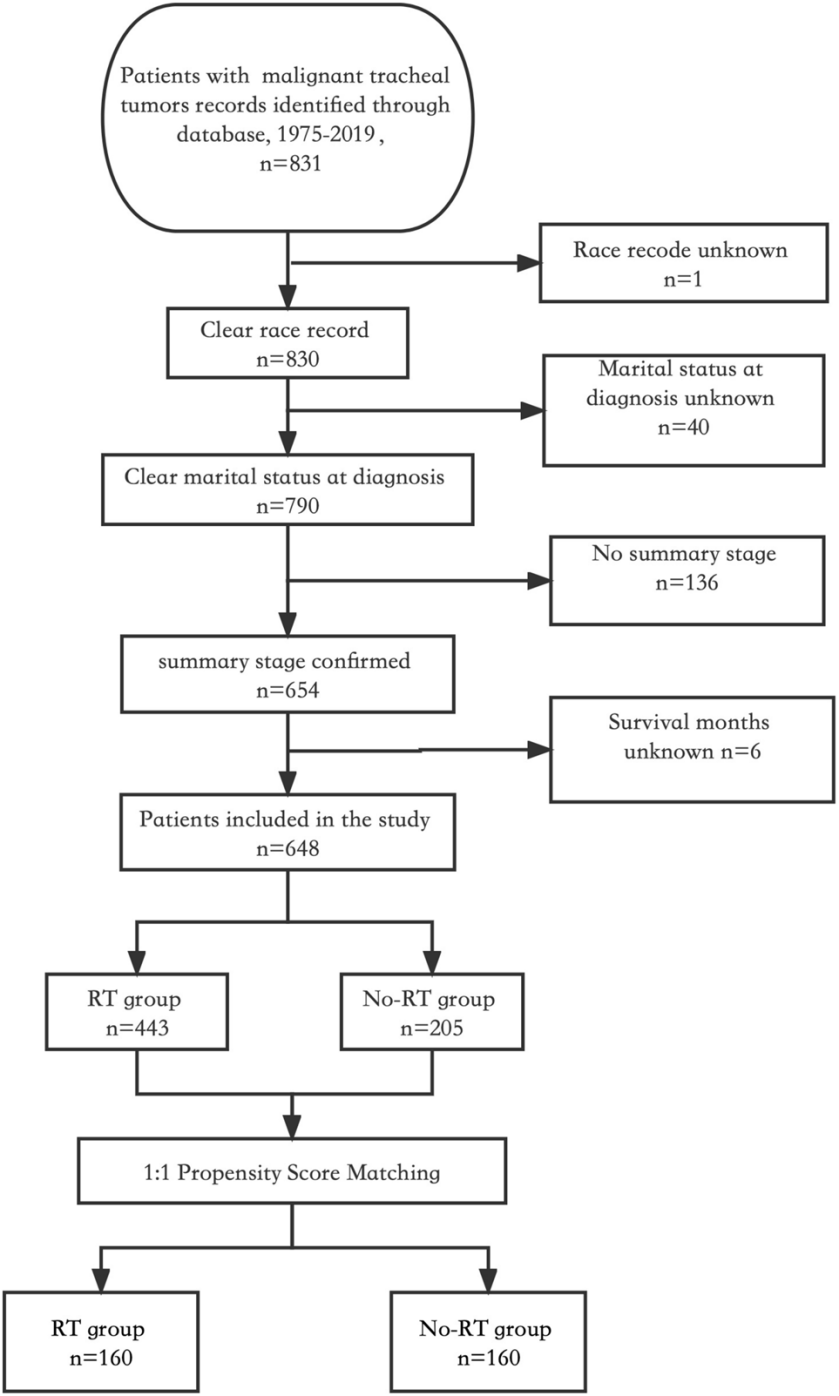
Flow chart depicting the patient selection process. RT: radiotherapy

**Fig. 2:**
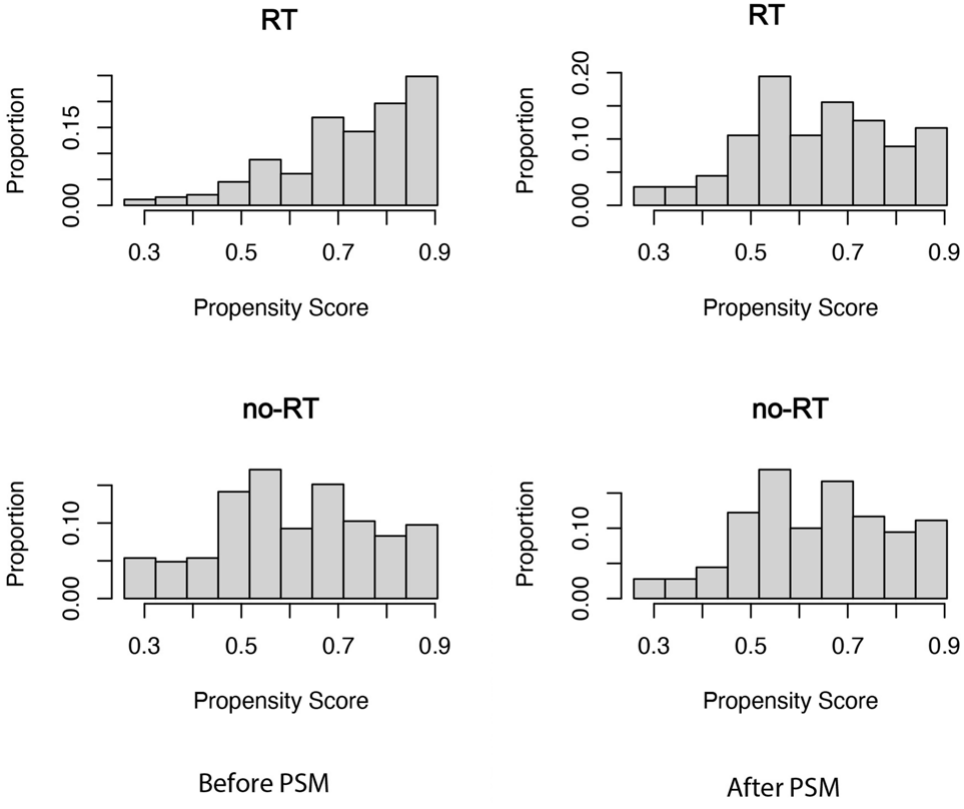
Propensity score distribution between RT and no-RT groups before and after propensity score matching

The study cohort included a total of 648 patients diagnosed with tracheal cancer and with complete information in the database. Among them, 443 (68.4%) underwent RT while the remaining 205 (31.6%) did not (Fig. 1). According to the study, the median age at diagnosis was 64 years, and there was a male to female ratio of 1.4:1. Following propensity score matching, both groups consisted of 160 patients each, with an average propensity score of 0.63 ± 0.15 (Fig. 2).

Table 1 shows that without PSM, there were significant imbalances in baseline demographic and clinical characteristics between the two groups. However, after PSM, the covariates were well balanced between the groups, indicating that PSM was successful in minimizing potential confounds. Overall, PSM helped to increase the comparability of the study groups.

**Table 1:** Baseline characteristics before and after propensity score matching, showing statistical comparisons between RT and no-RT groups

Factor	Pre-PSM			Post-PSM		
	RT	No-RT	*P*	RT	No-RT	*P*
	*n* = 443	*n* = 205		*n* = 180	*n* = 180	
Age (*n*, %)						
≤ 60	261	77	0.40	74	69	0.67
> 60	182	128		106	111	
Ethnicity (*n*, %)						
White	362	166	0.91	138	150	0.11
Other	81	39		42	30	
Marital status (*n*, %)						
Married	269	125	0.95	110	106	0.67
Other	174	80		70	74	
Diagnosis year (*n*, %)						
> 1994	220	115	0.15	100	99	0.92
≤ 1994	223	90		80	81	
Pathology (*n*, %)						
Adenocarcinoma	106	45	< 0.001	37	40	0.76
Squamous cell carcinoma	254	87		91	84	
Other	83	73		52	56	
Disease extension (*n*, %)						
Local	136	114	< 0.001	92	92	0.47
Regional	217	60		49	57	
Distant	90	31		39	31	
Sex (*n*, %)						
Female	192	79	0.25	74	68	0.52
Male	251	126		106	112	
Surgery (*n*, %)						
Yes	260	89	< 0.001	97	95	0.83
No	183	116		83	85	

### Survival outcomes after PSM

**Fig. 3:**
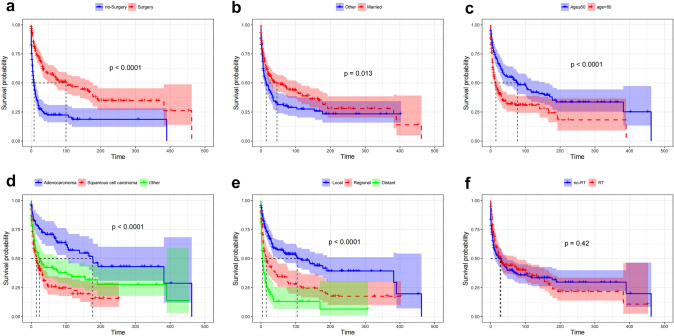
Kaplan–Meier overall survival (OS) estimates and 95% confidence intervals for patients in surgery and no-surgery groups: **a** in the total sample, grouped by **b** marital status, **c** age, **d** pathology, **e** disease extension, **f** radiotherapy

According to the study, we investigated the prognostic impact of RT by comparing the median overall survival between PS-matched groups, with each group consisting of 160 patients. Univariate logistic analysis of eight potential factors revealed four related variables, including sex, marital status, race, pathology, diagnosis year, disease extension, age, and surgery (Table 2). The results demonstrated that surgery was a significant predictor of overall survival, with a median overall survival of 8–12 months for patients receiving surgery and 4–7 months for patients in the no-surgery group (log-rank test: *χ*^21^ = 34.0, *P* < 0.001, Fig. 3a). Additionally, married patients (log-rank test: *χ*^21^ = 6.2, *P* < 0.013, Fig. 3b) and those aged ≤ 60 years (log-rank test: *χ*^21^ = 15.7, *P* < 0.001, Fig. 3c) had a significantly better overall survival compared to unmarried patients and those aged > 60 years, respectively. Patients with different types of pathology (log-rank test: *χ*^21^ = 25.4, *P* < 0.001; Fig. 3d) and tumor extension (log-rank test: *χ*^21^ = 59.7, *P* < 0.001; Fig. 3e) also had a tendency for different overall survival rates. Despite investigating the prognostic impact of RT, the study found no significant difference in median overall survival between the RT group and the no-RT group (*P* = 0.42; Fig. 3f).

**Table 2:** Univariate and multivariate logistic analyses of patients with tumors in the trachea

Factor	*N*	Median OS (months)	Univariate *P*	Multivariate	
				HR (95% CI)	*P*
Age				1.509 (1.125–2.024)	0.006
≤ 60	143	77	< 0.001		
> 60	217	14			
Ethnicity					
White	288	20	0.497		
Other	72	46			
Marital status				0.681 (0.518–0.898)	0.007
Married	216	46	0.013		
Other	144	15			
Diagnosis year					
> 1994	199	35	0.060		
≤ 1994	161	20			
Pathology					
Adenocarcinoma	77	177	< 0.001	Reference	
Squamous cell carcinoma	175	15		1.893 (1.257–2.848)	0.002
Other	108	24		1.253 (0.812–1.932)	0.308
Disease extension					
Local	184	104	< 0.001	Reference	
Regional	106	15		1.860 (1.349–2.564)	< 0.001
Distant	70	4		2.778 (1.938–3.981)	<0.001
Sex					
Female	142	29	0.642		
Male	218	26			
Surgery				0.479 (0.354–0.646)	< 0.001
Yes	192	100	< 0.001		
No	168	8			
Radiotherapy					
Yes	160	29	0.422		
No	160	27

**Fig. 4:**
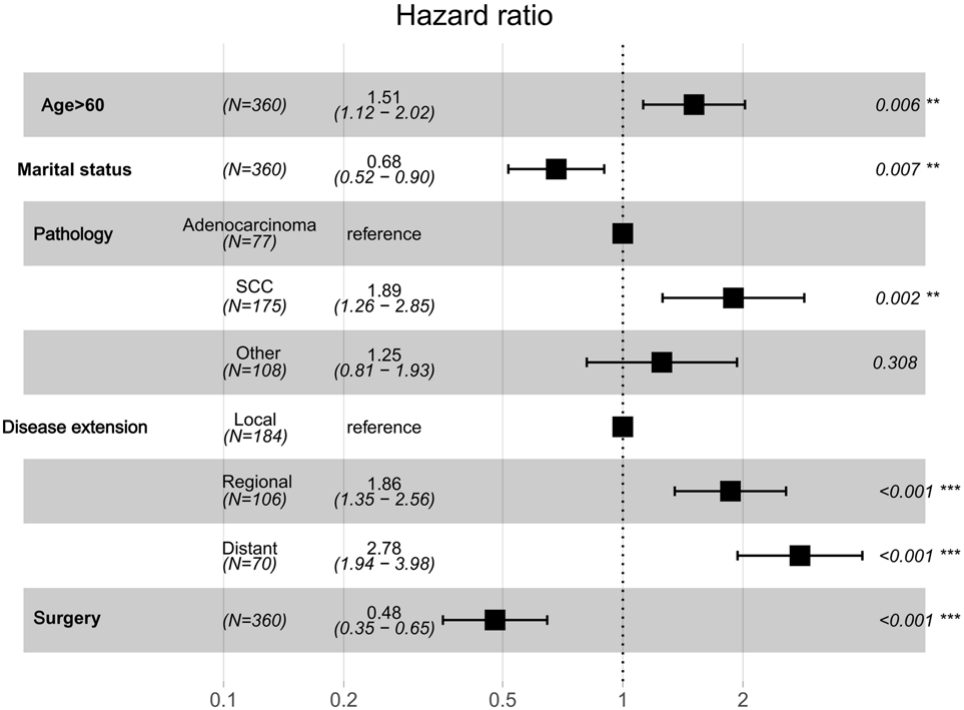
Cox proportional hazard ratios with 95% confidence intervals. There is a dashed line for an equivalent hazard ratio (HR 1)

**Fig. 5:**
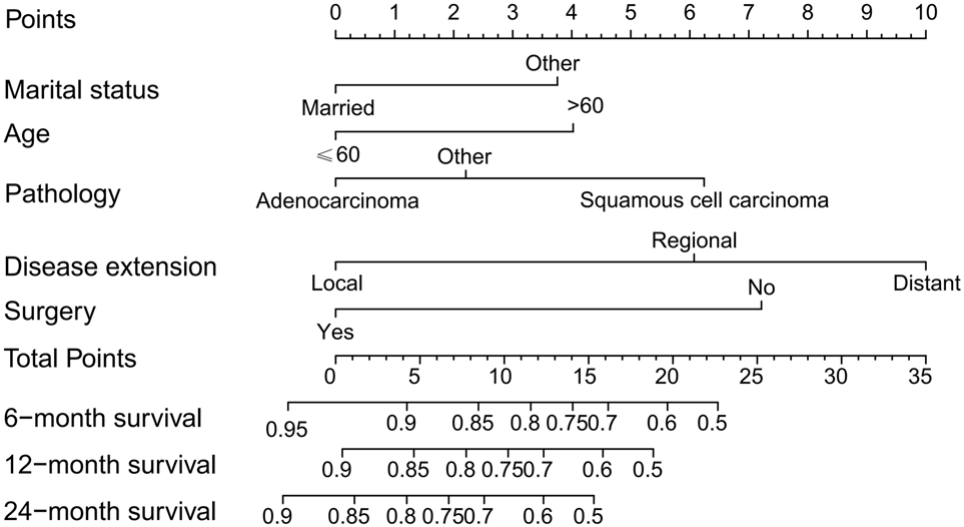
A nomogram to estimate the risk of patients with malignant tumors in the trachea

The multivariate logistic regression analysis confirmed age, marital status, disease extension, and surgery as independent risk predictors in patients with malignant tracheal tumors (Table 2; Fig. 4). A Cox proportional hazards model was utilized to create a nomogram that incorporates key variables, including age, marital status, disease extension, pathology, and surgical intervention, which predicts 6-, 12-, and 24-month overall survival rates (Fig. 5). The model’s discrimination ability was assessed using a C-index of 0.775. Overall, the results indicate that surgery is an important predictor of overall survival in patients with malignant tracheal tumors, and the nomogram could potentially be used as a tool for prognostic prediction in clinical practice.

### Analysis of the subsets

**Fig. 6:**
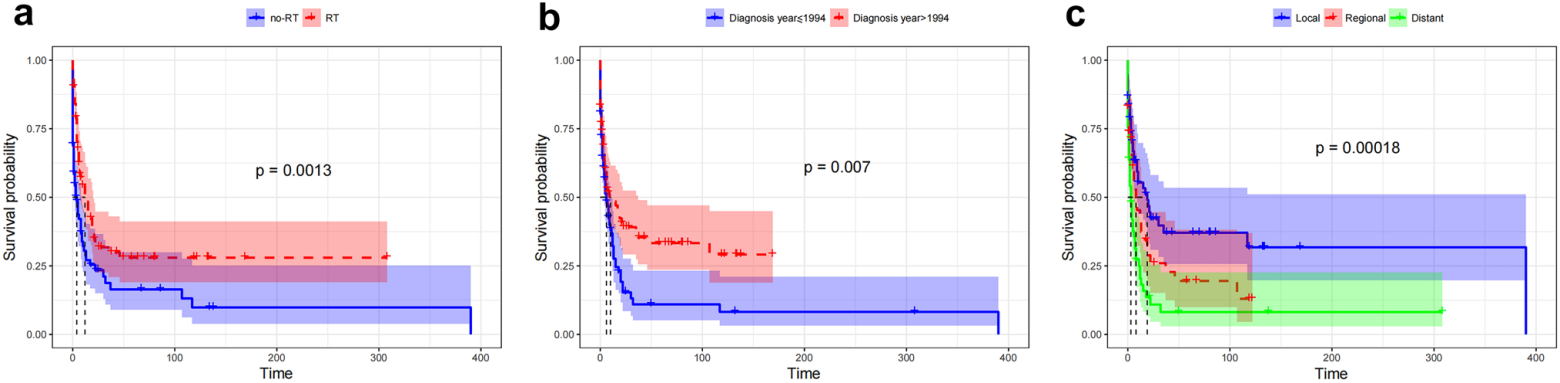
Kaplan–Meier overall survival (OS) estimates and 95% confidence intervals for patients without surgery in RT and no-RT groups: **a** grouped by **b** diagnosis year, **c** disease extension

The analysis investigated whether radiotherapy is an effective treatment option for patients who are no longer eligible for surgery. Patients who received RT had better overall survival compared to those who did not receive RT (median survival of 12 months vs. 4 months, respectively, *P* = 0.001, Fig. 6a). Furthermore, the analysis indicated that the year of disease diagnosis and tumor extension were significant prognostic factors for overall survival in patients who did not receive surgical treatment. Specifically, patients diagnosed after 1994 demonstrated improved overall survival compared to those diagnosed in, or prior to, 1994 (median survival of 10 months vs. 6 months, respectively, *P* = 0.007, Fig. 6b), and patients with tumor local extension had better overall survival compared to those with tumor regional or distant extension (median survival of 19 months vs. 8 months vs. 3 months, respectively, *P* < 0.0001, Fig. 6c).

A multivariable Cox analysis was carried out to investigate the independent factors associated with the subset of patients who did not receive surgical intervention. The analysis revealed that RT and disease extension were independent predictors for prognosis. Patients who received RT had a lower risk of mortality compared to those who did not receive RT (HR 0.567, *P* = 0.003), while those with more extensive disease were at higher risk of mortality (HR 0.446, *P* < 0.0001).

Overall, these findings indicate that RT could be a compelling therapeutic option for patients who are contraindicated for surgical intervention. Moreover, tumor extension and the year of disease diagnosis are critical prognostic factors for predicting overall survival.

## Discussion and conclusion

Tracheal cancers are uncommon malignancies, and there is a scarcity of large-scale studies to provide guidance on their management. However, preliminary investigations demonstrate that radiation therapy may improve overall survival and decrease the incidence of tracheal cancer-related mortality in patients suffering from primary malignant tracheal tumors who are medically unsuitable for surgery. Surgery is regarded as one of the best treatment options for these tumors; however, its implementation is restricted by factors such as the tumor’s size, location, and extent, as well as the presence of comorbidities. While radiation therapy is an important local treatment modality, its efficacy in treating malignant tracheal tumors remains contentious. Neoadjuvant radiation therapy is not recommended, as it can hinder bronchial blood supply, causing delays in anastomotic site healing, and increasing the risk of dehiscence, especially in patients with invasive extraluminal tracheal tumors that require comprehensive treatment. Nevertheless, a study conducted by Napieralska and colleagues confirmed the potential of radiation therapy in treating malignant tracheal tumors (Napieralska et al. 2016). In a retrospective analysis of 58 cases of pathologically confirmed malignant tracheal tumors, Napieralska et al. found that radiation therapy (*P* = 0.013), performance status (PS) score (*P* = 0.033), and hemoptysis (*P* = 0.003) were independent prognostic factors that were associated with greater longterm survival in patients with tracheal malignancies. Local surgical or endoscopic resection therapy was only found to be a survival prognostic factor in the univariate analysis (*P* = 0.030); however, in the multivariate analysis, its impact on survival was not statistically significant (*P* = 0.324). Larger studies on surgical treatment of tracheal tumors indicate that 70% of patients are eligible for surgical resection, while 70% of patients with adenoid cystic carcinoma of the trachea require postoperative radiation therapy (Grillo and Mathisen 1990). For incompletely resected moderately to poorly differentiated malignant tumors, adjuvant radiation therapy is recommended after surgery (Fields et al. 1989). Je et al. believe that for adenoid cystic carcinoma of the trachea that has undergone surgical resection with residual lesions found under the microscope, adjuvant radiotherapy should be given (Je et al. 2017).

The management of unresectable primary tracheal tumors is not well-defined due to the scarcity of reliable data. However, for patients who are not eligible or decline surgical intervention, radical radiotherapy should be administered whenever feasible. This approach can provide a viable alternative to surgical resection, and may significantly improve the prognosis for patients with primary tracheal tumors that cannot be surgically removed (Graham 1997; Thotathil et al. 2004). Histology is a significant determinant in determining the outcome of radical radiotherapy, and patients with adenoid cystic carcinoma tend to have better prognoses than those afflicted with SCC (Fields et al. 1989). For adenoid cystic carcinoma of the trachea that cannot be completely resected surgically, definitive radiotherapy is an effective treatment (Je et al. 2017). Chen et al. also found that adjuvant radiotherapy significantly prolonged progression-free survival (PFS) (*P* = 0.027) and OS (*P* = 0.004) in 24 cases of incompletely resected tracheal adenoid cystic carcinoma (Chen et al. 2015). Tracheal squamous cell carcinoma has a relatively poor prognosis, and its radio-sensitivity is generally considered inferior to that of tracheal adenoid cystic carcinoma. Tracheal Squamous cell carcinoma, on the other hand, generally has a poor prognosis and is considered to be less responsive to radiotherapy than adenoid cystic carcinoma. However, SCC managed with radiotherapy in other locations has demonstrated favorable outcomes in terms of local control and survival, including in the postoperative setting among patients with lung, cervix, head, and neck cancers (Lung Cancer Study 1986; Peters et al. 1993; Rotman et al. 2006; Trodella et al. 2002). A retrospective analysis conducted by Xie et al. on 78 patients who received radiotherapy and 78 patients who did not receive radiotherapy for tracheal SCC from the SEER database showed that overall survival was significantly prolonged in patients who received radiotherapy, with a decreased cumulative mortality rate(Xie et al. 2012).

Due to the low incidence of malignant tumors in the trachea, research on the doseresponse relationship of radiotherapy is limited. It is common to administer a dose of 54 Gy for tracheal tumors, which can be increased up to 60 Gy with the use of intensity-modulated radiotherapy (IMRT). Mornex et al. suggested that the dose of radiotherapy is a prognostic factor for primary tracheal tumors. The 5-year survival rate of patients who received > 56 Gy of radiotherapy was 12%, while that of patients who received low-dose radiotherapy dropped to 5% (Mornex et al. 1998). Levy et al. performed a retrospective analysis of 31 cases of tracheal adenoid cystic carcinoma that were treated at their center from 1984 to 2014. They found that whether the radiotherapy dose exceeded 60 Gy was an independent prognostic factor for progression-free survival (Levy et al. 2018). Similar findings were reported by Licht et al., showing that tracheal malignancies treated with a radiation dose over 60 Gy had a 2-year survival rate superior to that of patients receiving less than 40 Gy (Licht et al. 2001). High-dose radiotherapy may increase local tumor control rates and survival time. However, it also increases the risk of complications, such as tracheobronchial fistulas, airway stenosis, and tracheal necrosis (Alraiyes et al. 2013; Kelsey et al. 2006; Miller et al. 2005). With the advancement of radiotherapy techniques, complications after high-dose radiotherapy can be minimized. However, there is currently a lack of randomized trials on curative radiotherapy for primary tracheal tumors, and the optimal dose and fractionation of radiotherapy remain uncertain.

This analysis has several limitations that should be considered. Firstly, retrospective studies and population-based studies have inherent limitations that could affect the reliability and validity of the results. Secondly, the sample size of patients with malignant tumors of the trachea (*N* = 360) is relatively small, which could have contributed to potential errors in the analysis. Thirdly, there are numerous factors, including tumor size and systemic treatments, that could not be taken into account due to the limitations of the SEER registry. Finally, while race does not seem to impact the prognosis of patients with malignant tumors of the trachea, it is important to note that the majority of study participants were white, so the generalizability of these models to other ethnic groups is uncertain and necessitates further investigation.

Our study has found that surgery, marital status, disease extension, pathology, and age are all independent risk factors for patients diagnosed with tracheal malignant tumors. For patients who did not undergo surgery, radiotherapy and disease extension were identified as independent prognostic factors. The development of nomograms as an intuitive graphic tool can facilitate the quantitative evaluation of risk and prognosis, and guide clinical decision-making in cases of tracheal malignant tumors.

## Data Availability

The datasets generated during and/or analyzed during the current study are available from the corresponding author on reasonable request.

## Abbreviations

PTMT: Primary tracheal malignant tumors
RT: Radiotherapy
SEER: Surveillance, epidemiology, and end results
PSM: Propensity score matching
SCC: Squamous cell carcinoma
ACC: Adenoid cystic carcinoma
OS: Overall survival
HR: Hazard rate
PFS: Progression-free survival
IMRT: Intensity-modulated radiotherapy

## Data availability

The original contributions can be downloaded from the SEER database. Further inquiries can be directed to the corresponding authors.

## References

[CR1] Alraiyes AH, Alraies MC, Abbas A (2013). Radiation-associated airway necrosis. Ochsner J.

[CR2] Austin PC (2009). Balance diagnostics for comparing the distribution of baseline covariates between treatment groups in propensity-score matched samples. Stat Med.

[CR3] Austin PC (2010). Statistical criteria for selecting the optimal number of untreated subjects matched to each treated subject when using many-to-one matching on the propensity score. Am J Epidemiol.

[CR4] Chen F, Huang M, Xu Y, Li T, Xie K, Zhang L, Lu Y (2015). Primary tracheal adenoid cystic carcinoma: adjuvant treatment outcome. Int J Clin Oncol.

[CR5] Chow DC, Komaki R, Libshitz HI, Mountain CF, Ellerbroek N (1993). Treatment of primary neoplasms of the trachea. The role of radiation therapy. Cancer.

[CR6] Fields JN, Rigaud G, Emami BN (1989). Primary tumors of the trachea. Results of radiation therapy. Cancer.

[CR7] Graham MV, Emani B, Perez CA, Brady LW (1997). Mediastinum and trachea [M]. Principles and practice of radiation oncology.

[CR8] Grillo HC, Mathisen DJ (1990). Primary tracheal tumors: treatment and results. Ann Thorac Surg.

[CR9] Honings J, van Dijck JA, Verhagen AF, van der Heijden HF, Marres HA (2007). Incidence and treatment of tracheal cancer: a nationwide study in the Netherlands. Ann Surg Oncol.

[CR10] Honings J, Gaissert HA, Verhagen AF, van Dijck JA, van der Heijden HF, van Die L, Marres HA (2009). Undertreatment of tracheal carcinoma: multidisciplinary audit of epidemiologic data. Ann Surg Oncol.

[CR12] Je HU, Song SY, Kim DK, Kim YH, Jeong SY, Back GM, Choi EK (2017). A 10-year clinical outcome of radiotherapy as an adjuvant or definitive treatment for primary tracheal adenoid cystic carcinoma. Radiat Oncol.

[CR13] Kelsey CR, Kahn D, Hollis DR, Miller KL, Zhou SM, Clough RW, Marks LB (2006). Radiation-induced narrowing of the tracheobronchial tree: an in-depth analysis. Lung Cancer.

[CR14] Levy A, Omeiri A, Fadel E, Le Pechoux C (2018). Radiotherapy for tracheal-bronchial cystic adenoid carcinomas. Clin Oncol (r Coll Radiol).

[CR15] Licht PB, Friis S, Pettersson G (2001). Tracheal cancer in Denmark: a nationwide study. Eur J Cardiothorac Surg.

[CR16] Lung Cancer Study G (1986). Effects of postoperative mediastinal radiation on completely resected stage II and stage III epidermoid cancer of the lung. N Engl J Med.

[CR17] Manninen MP, Antila PJ, Pukander JS, Karma PH (1991). Occurrence of tracheal carcinoma in Finland. Acta Otolaryngol.

[CR18] Miller KL, Shafman TD, Anscher MS, Zhou SM, Clough RW, Garst JL, Marks LB (2005). Bronchial stenosis: an underreported complication of high-dose external beam radiotherapy for lung cancer?. Int J Radiat Oncol Biol Phys.

[CR19] Mornex F, Coquard R, Danhier S, Maingon P, El Husseini G, Van Houtte P (1998). Role of radiation therapy in the treatment of primary tracheal carcinoma. Int J Radiat Oncol Biol Phys.

[CR20] Napieralska A, Miszczyk L, Blamek S (2016). Tracheal cancer—treatment results, prognostic factors and incidence of other neoplasms. Radiol Oncol.

[CR21] Nouraei SM, Middleton SE, Nouraei SA, Virk JS, George PJ, Hayward M, Sandhu GS (2014). Management and prognosis of primary tracheal cancer: a national analysis. Laryngoscope.

[CR22] Peters LJ, Goepfert H, Ang KK, Byers RM, Maor MH, Guillamondegui O (1993). Evaluation of the dose for postoperative radiation therapy of head and neck cancer: first report of a prospective randomized trial. Int J Radiat Oncol Biol Phys.

[CR23] Rotman M, Sedlis A, Piedmonte MR, Bundy B, Lentz SS, Muderspach LI, Zaino RJ (2006). A phase III randomized trial of postoperative pelvic irradiation in Stage IB cervical carcinoma with poor prognostic features: follow-up of a gynecologic oncology group study. Int J Radiat Oncol Biol Phys.

[CR24] Thotathil ZS, Agarwal JP, Shrivastava SK, Dinshaw KA (2004). Primary malignant tumors of the trachea—the Tata Memorial Hospital experience. Med Princ Pract.

[CR25] Trodella L, Granone P, Valente S, Valentini V, Balducci M, Mantini G, Cellini N (2002). Adjuvant radiotherapy in non-small cell lung cancer with pathological stage I: definitive results of a phase III randomized trial. Radiother Oncol.

[CR26] Urdaneta AI, Yu JB, Wilson LD (2011). Population based cancer registry analysis of primary tracheal carcinoma. Am J Clin Oncol.

[CR27] Xie L, Fan M, Sheets NC, Chen RC, Jiang GL, Marks LB (2012). The use of radiation therapy appears to improve outcome in patients with malignant primary tracheal tumors: a SEER-based analysis. Int J Radiat Oncol Biol Phys.

